# PEDV infection downregulates goblet cell differentiation through activating the Notch pathway

**DOI:** 10.1186/s13567-025-01599-5

**Published:** 2025-08-12

**Authors:** Yi Wang, Shanshan Yang, Yongxiang Zhao, Shuo Tian, Qiuxia Cao, Xinmei Geng, Mengdi Yang, Xu Song, Hongqi Shang, Shiyu Liu, Rongli Guo, Yunchuan Li, Min Sun, Mi Hu, Baochao Fan, Bin Li

**Affiliations:** 1 School of Veterinary Medicine, Hebei Agricultural University, Baoding, China; 2 Institute of Veterinary Medicine, Key Laboratory of Veterinary Biological Engineering and Technology, Ministry of Agriculture, Jiangsu Key Laboratory for Food Quality and Safety-State Key Laboratory Cultivation Base of Ministry of Science and Technology, Jiangsu Academy of Agricultural Sciences, Nanjing, China; 3 College of Veterinary Medicine, Nanjing Agricultural University, Nanjing, 210095 China; 4 School of Life Sciences, Jiangsu University, Zhenjiang, China; 5 School of Food and Biological Engineering, Jiangsu University, Zhenjiang, China; 6 Jiangsu Co-Innovation Center for Prevention and Control of Important Animal Infectious Diseases and Zoonoses, Yangzhou University, Yangzhou, China; 7 GuoTai (Taizhou) Center of Technology Innovation for Veterinary Biologicals, Taizhou, China

**Keywords:** PEDV, Notch signalling pathway, goblet cells, ORF3, intestinal organoids

## Abstract

Porcine epidemic diarrhoea virus (PEDV) is the most widespread porcine coronavirus worldwide, causing high mortality and a high incidence rate among piglets. The molecular mechanisms by which PEDV regulates epithelial cell function and differentiation, as well as its disruption of the intestinal mucosal barrier, are not yet fully understood. This study reveals that PEDV infection reduces the number of goblet cells and impairs the intestinal barrier integrity in newborn piglets. Regarding the pathways involved in the differentiation of intestinal stem cells (ISCs), PEDV infection concurrently activates the Notch and MAPK pathways while suppressing the Wnt/β-catenin pathway in the intestines of piglets. Furthermore, in vitro experiments using intestinal monolayer organoid models showed that PEDV infection hinders goblet cell differentiation by activating the Notch signalling pathway. Additionally, the PEDV-encoded ORF3 protein, which is crucial for activating the Notch pathway, inhibits goblet cell differentiation in PEDV-infected intestinal monolayer organoids. This study offers new insights into the mechanisms underlying intestinal mucosal barrier dysfunction induced by PEDV infection.

**Supplementary Information:**

The online version contains supplementary material available at 10.1186/s13567-025-01599-5.

## Introduction

Coronaviruses pose a significant threat to both human and animal health due to their high pathogenicity [1]. In swine, diarrhoea can result from various pathogens, with the coronavirus of porcine epidemic diarrhoea virus (PEDV) being the primary cause [2, 3]. This highly contagious virus leads to acute enteritis in piglets, characterised by severe vomiting, watery diarrhoea, and extreme dehydration [4, 5]. It is particularly devastating for suckling piglets, where the mortality and morbidity rates can reach as high as 80% to 100% [6]. As the most common porcine coronavirus, PEDV has spread globally, resulting in substantial economic losses for the swine industry [7].

The PEDV genome is approximately 28 kilobases (kb) long and contains seven open reading frames (ORFs) that encode both nonstructural and structural proteins. Specifically, ORF1a/1b produce non-structural proteins, while the spike (S), envelope (E), membrane (M), and nucleocapsid (N) genes encode the four principal structural proteins [8].

Between the S and E genes, ORF3 encodes the only accessory protein of PEDV [9, 10]. ORF3 is known to have ion channel activity and features multiple transmembrane domains, which play a role in regulating viral replication and virulence [11]. Furthermore, PEDV ORF3 inhibits apoptosis and promotes viral autophagy to enhance viral replication [12, 13].

The intestinal mucosal barrier is a complex defence mechanism that is composed of mucosal epithelial cells, the mucosal immune system, and gut microbiota [14]. Epithelial cells are crucial components of this barrier, maintaining their function by forming tight junctions (TJs) and producing mucin [15]. As they migrate along the crypt-villus axis, epithelial cells differentiate into specialised mature cells, such as absorptive enterocytes and secretory cells [16]. Goblet cells, a specific type of secretory epithelial cell, play a vital role in preserving the integrity of the intestinal mucosal barrier. They not only synthesise and secrete mucin (MUC) family proteins but also produce various antibacterial proteins.

Recent studies on coronaviruses affecting the intestinal tract have shown that transmissible gastroenteritis virus (TGEV) infection specifically targets Paneth cells, which impairs the ability of Lgr5 + intestinal stem cells (ISCs) to differentiate into goblet cells [17]. Additionally, porcine delta coronavirus (PDCoV) infection activates the Notch signalling pathway, which suppresses goblet cell differentiation and reduces mucus production [18]. However, further research is needed to understand how PEDV infection modulates the intestinal Notch pathway.

Intestinal epithelial homeostasis relies on the regenerative capacity of ISCs located at the crypt base [19]. These ISCs give rise to two main cell lineages: secretory cells (such as Paneth cells, goblet cells, enteroendocrine cells, and tuft cells) and absorptive enterocytes [20, 21].

The Notch signalling pathway plays a crucial role in regulating important cellular processes, such as the proliferation and differentiation of ISCs [22]. Notch signalling influences the fate of secretory cells by inhibiting the basic helix-loop-helix (bHLH) transcription factor ATOH-1 [23]. Moreover, Notch activation also induces HES-1, which acts as a transcriptional repressor that suppresses ATOH-1 transcription and inhibits the differentiation of secretory cells. Notch ligands can activate Notch signalling in neighbouring cells, which further downregulates ATOH-1 and promotes the differentiation of absorptive cells [24].

In addition to the Notch pathway, the MAPK and Wnt/β-catenin pathways also regulate ISC differentiation. MAPK signalling modulates the choice between goblet and Paneth cell fates by regulating Wnt/β-catenin activity. Inhibition of the MAPK pathway increases Wnt/β-catenin signalling and promotes characteristics of Paneth cells, while high MAPK activity or inhibition of Wnt signalling tends to favour goblet cell properties [25].

This study demonstrates that PEDV infection in neonatal piglets leads to intestinal barrier dysfunction and impairs the differentiation of ISCs. After PEDV infection, we observed a reduction in the number of goblet cells, decreased mucus production, and damage to the intestinal mucosal barrier. Mechanistically, PEDV infection activates the Notch pathway, which inhibits the differentiation of ISCs into goblet cells. In summary, these findings indicate that PEDV compromises the intestinal mucosal barrier function by altering goblet cell differentiation and impairing mucus secretion.

## Materials and methods

### Virus

The PEDV AH2012/12 strain (Gene Bank number: KU646831.1) was isolated and stored in the laboratory [26].

### Animal experiments

Twelve PEDV-negative neonatal piglets were randomly assigned to four groups: a control group, a 6 hours post-infection (hpi) group, a 12 hpi group, and a 24 hpi group. The piglets are housed individually in incubators and fed milk every 4 h. Those in the PEDV challenge groups were orally administered 1 × 10^4^ TCID_50_ of PEDV, while the mock-infected piglets received an equal volume of phosphate-buffered saline (PBS) orally. Clinical symptoms, including vomiting, severe diarrhoea, and lethargy, were recorded prior to euthanasia. Small intestinal tissues were then collected for subsequent analyses, including western blotting (WB), haematoxylin and eosin (H&E) staining, immunohistochemistry (IHC), and periodic acid-Schiff (PAS) staining.

### The isolation of porcine intestinal crypts and the subsequent culture of three-dimensional (3D) enteroids

Intestinal crypts were isolated from specific pathogen-free (SPF) piglets aged 7 to 10 days using a modified protocol based on the method described by van der Hee et al. [27]. The intestinal tissue was longitudinally opened, sectioned into 2 mm pieces, and rinsed until the solution became clear. The fragments were then digested with Gentle Cell Dissociation Reagent (STEMCELL Technologies, Canada) to obtain the crypts.

The resulting crypt foci particles were resuspended in 10 mL of ice-cold PBS buffer containing 0.1% bovine serum albumin (BSA) and a penicillin–streptomycin solution. This suspension was filtered through a 70 μm cell strainer to remove any tissue debris. The crypt pellets were collected by centrifugation at 400 × *g* for 5 min at 4 ℃ and resuspended in 10 mL of ice-cold DMEM/F12 medium.

After counting the cells, the crypts were mixed with Corning^®^ Organoid Growth Medium (Corning, USA) and Matrigel Matrix Gel (BD Biosciences, USA) at specified ratios. The crypts were then inoculated in 24-well plates at a density of 200 crypts per well and placed in a 37 ℃, 5% CO_2_ incubator for three-dimensional culture. The plates were incubated at 37 ℃ for 10 min to allow the Matrigel to solidify, after which 800 μL of IntestiCult Organoid Growth Medium was added to each well. The cultures were maintained at 37 ℃ with 5% CO₂, with medium changes occurring every 3 to 5 days.

### Two-dimensional (2D) monolayer culture of intestinal enteroids

Following a 7 day culture period of 3D intestinal samples, precooled DMEM/F12 medium was added. The samples were collected and transferred to a 15 mL centrifuge tube, then centrifuged at 400 × *g* at 4 ℃ for 5 min. The pelleted enteroids were enzymatically digested at 37 ℃ using 0.25% trypsin–EDTA (Gibco) for 5 min. This was followed by mechanical disruption through repeated pipetting to create single-cell suspensions. The suspensions were then neutralised with DMEM/F12 medium containing 20% fetal bovine serum (FBS) and centrifuged again (400 × *g*, 5 min). The cells were resuspended in IntestiCult Organoid Growth Medium at room temperature (RT) and plated at a density of 200 cells per well in Matrigel-coated 48-well plates. After three days of differentiation, 2D monolayers of the intestinal enteroids were obtained for subsequent assays.

### Western blotting (WB)

Intestinal tissue samples were first homogenised in liquid nitrogen and then placed in radioimmunoprecipitation assay (RIPA) buffer (Beyotime, China), which contained a protease inhibitor mixture (PMSF). The samples were lysed at 4 ℃ for 10 min. After lysis, the samples were centrifuged at 12000 rpm for 3–5 min at 4 ℃, and the supernatant was collected.

The total protein concentration was determined using a bicinchoninic acid (BCA) assay kit (Beyotime, China). For analysis, 20 µg of protein was taken from each sample and separated by electrophoresis through a 10% sodium dodecyl sulfate–polyacrylamide gel electrophoresis (SDS-PAGE) system. The separated proteins were then transferred to a polyvinylidene difluoride (PVDF) membrane.

The membranes were blocked with 5% skim milk in PBS with Tween 20 (PBST) for 1 h, after which they were incubated with primary antibodies at 4 ℃ overnight. The primary antibodies used included: anti-PEDV-N (1:2000, made in the laboratory), anti-ERK1/2 (1:1000, Beyotime, China), anti-phospho-ERK1/2 (1:1000, Beyotime, China), anti-β-catenin (1:1000, Proteintech, USA), anti-GAPDH (1:10000, Proteintech, USA), anti-HES-1 (1:500 dilution, ABclonal, China) and anti-ATOH-1 (1:5000, Proteintech, USA).

Following the primary antibody incubation, the membranes were washed three times with PBST and then incubated with HRP-labelled secondary antibodies (goat anti-rabbit or anti-mouse, 1:10000, ABclonal, China) for 1 h at room temperature. Finally, the protein bands were detected using an enhanced chemiluminescence (ECL) detection kit (ABclonal, China), and images were captured with a Tanon^™^ 5200 CE Chemi-Image System (Tanon, Shanghai, China).

### Tissue staining

Paraffin-embedded intestinal tissues were sectioned into 5 μm slices. Following dewaxing in xylene, the samples were gradually rehydrated through a descending ethanol series. Histological analysis was performed using an H&E staining kit (Solarbio, China) in accordance with the manufacturer’s protocols. Goblet cells were identified using PAS staining (Solarbio, China) following standard protocols.

### Virus infection

For 2D monolayer infection, cells were washed twice with PBS and then incubated with PEDV at an MOI of 1.0, along with 10 µg/mL of trypsin for 2 h. After the adsorption period, the unbound virus was removed by washing the cells twice with PBS. The infected cells were then maintained in IntestiCult organoid growth medium, supplemented with 50 µg/mL trypsin, at 37 °C, until harvest.

### Quantitative real-time PCR (RT-qPCR) analyses

Total RNA was extracted from the sample following the instructions provided in the FastPure Cell/Tissue Total RNA Isolation Kit V2 (Vazyme, China). The concentration and purity of the RNA were determined using a Nanodrop^™^ One spectrophotometer (Thermo Fisher Scientific, USA). Following this, 1 µg of total RNA was reverse transcribed into cDNA using the Hiscript II qRT supermax (Vazyme, China).

RT-qPCR was then conducted using the Applied Biosystems Q6 real-time PCR instrument (USA) by AceQ Universal SYBR qPCR Master Mix (Vazyme, China). To ensure data reliability, technical replicates were performed for each reaction. The relative expression of genes was calculated using the 2^−ΔΔCT^ method, with internal reference genes (such as GAPDH in this study are listed in Additional file 1.

### Immunohistochemistry (IHC)

In immunohistochemistry experiments, sections of the jejunum were fixed onto positively charged glass slides, followed by dewaxing and rehydration using a series of ethanol solutions. To restore antigen epitopes, the sections underwent steam-induced antigen retrieval for 20 min. After this, the sections were blocked with 10% normal goat serum for 1 h to minimise nonspecific binding. Primary antibodies against PEDV-N, HES-1, and ATOH-1 were then applied and incubated. Following this, fluorescently labelled secondary antibodies, including CY3-conjugated goat anti-mouse IgG and FITC-conjugated goat anti-rabbit IgG (Abcam, UK), were added and incubated at room temperature for 1 h. Finally, the expression and localisation of the target proteins were visualised using a fluorescence microscope.

### Immunofluorescence (IF) analyses

The procedure was as follows: First, cells were fixed with 4% paraformaldehyde (PFA) at room temperature for 30 min. Subsequently, the samples were permeabilised with 0.2% Triton X-100 to improve antibody penetration. Next, the samples were blocked using a blocking buffer containing PBS and 5% skim milk to minimise nonspecific binding.

After blocking, the samples were incubated with the primary antibody at 37 ℃ for 2 h, followed by incubation with a fluorescently labelled secondary antibody under the same conditions for 1 h. For the specific detection of intestinal epithelial cell markers, an anti-mucin 2 antibody (1:50 dilution, ABclonal, China) was used to label goblet cells. The fluorescently labelled secondary antibody (Proteintech, China) was incubated for 1 h, and DAPI (Solarbio, China) was used to stain the nuclei for 10 min.

Finally, the samples were thoroughly washed with PBS, and fluorescence signals were visualised using a Zeiss LSM880 confocal microscope.

### Statistical analysis

In this study, all experiments consisted of at least three independent biological replicates to ensure the reliability and reproducibility of the data. Data analysis was performed using GraphPad Prism software. Statistical methods employed included unpaired two-tailed t-tests and one-way analysis of variance (ANOVA) to assess the significance of differences between groups. The level of statistical significance was set at *p* < 0.05, with the following markers indicating significance: *indicates *p* < 0.05, **indicates *p* < 0.01, and ***indicates *p* < 0.001.

## Results

### PEDV infection reduces goblet cells in the small intestine villi of piglets

PEDV primarily affects the small intestine, especially the jejunum and ileum, leading to significant damage [14]. Histopathological analysis of jejunal tissues at 24 hpi, as demonstrated in Additional file 2, revealed villus shortening, crypt deepening, and a disruption of intestinal homeostasis. The increased expression of IFN-β, IFN-λ, and ISGs indicated an antiviral response. However, the downregulation of tight junction proteins (ZO-1, Occludin, and Claudin) compromised the integrity of the intestinal barrier. These findings confirm that PEDV induces villous atrophy, crypt hyperplasia, and barrier dysfunction.

The intestinal epithelium primarily consists of absorptive enterocytes and secretory cells, including goblet cells that produce mucin [28]. These goblet cells are crucial for maintaining the protective mucus layer and epithelial integrity [29].

**Figure 1:**
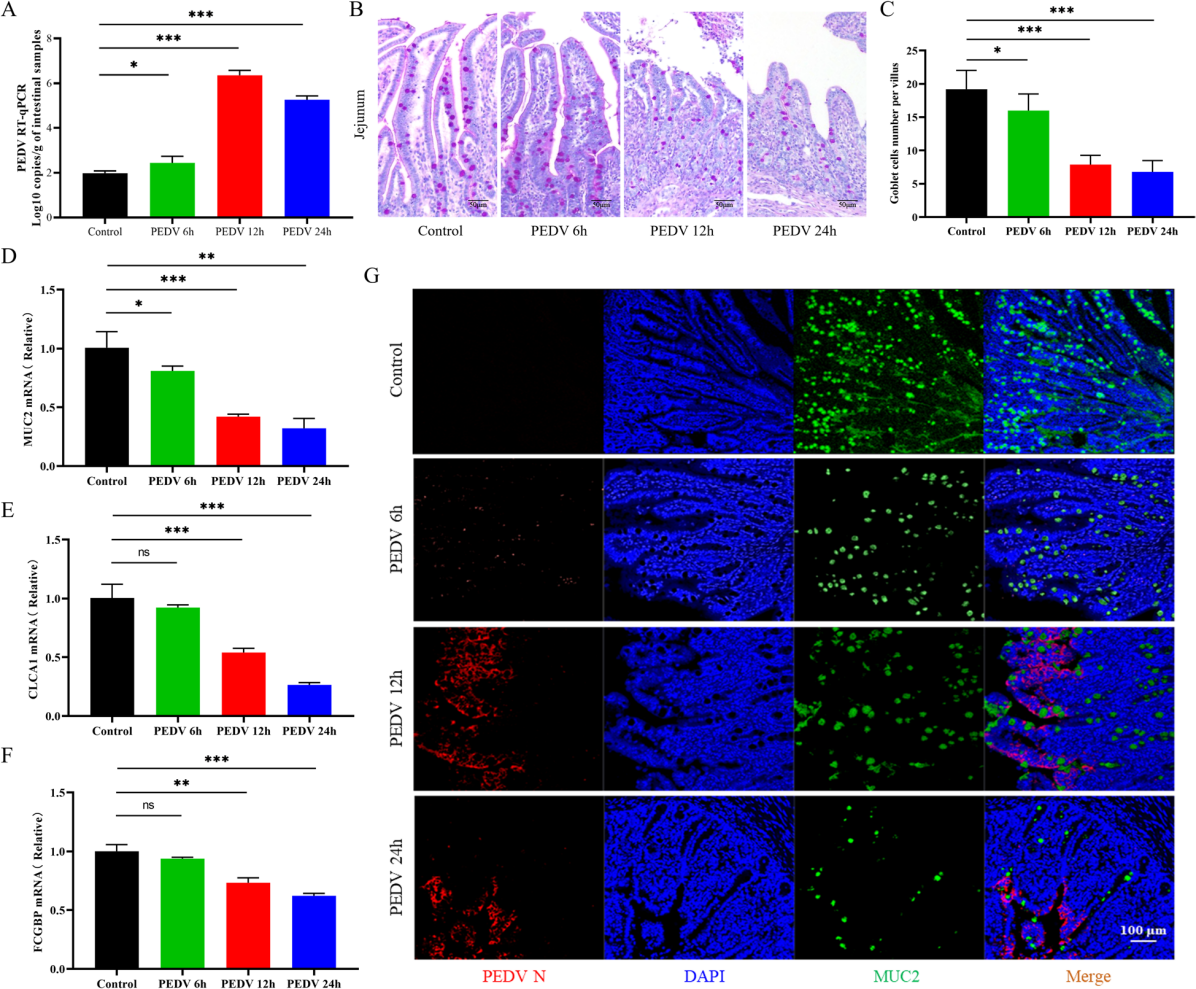
**The reduction of goblet cells in the intestinal villi of piglets infected with PEDV.** **A** Viral copy numbers in jejunal tissues of uninfected and PEDV-infected piglets at 6, 12, and 24 hpi. **B** Staining of goblet cells in the intestinal villi with PAS in jejunal tissues (purple). Scale bar = 50 μm. **C** The number of goblet cells per villus in the jejunum of control and PEDV-infected piglets at different time points. **D**–**F** mRNA levels of MUC2, CLCA1, and FCGBP in homogenised jejunum tissues. **G** MUC2 (green) and PEDV (red) positive cells in jejunum tissues. Scale bar = 100 μm. *p < 0.05, **p < 0.01, ***p < 0.001.

RT-qPCR results indicated that the viral load in the jejunal tissues of PEDV-infected piglets increased continuously from 6 to 24 hpi (Figure 1A). PAS analysis revealed a significant decrease in the number of goblet cells in the jejunum of PEDV-infected piglets (Figures 1B and C). Furthermore, mRNA analysis showed a notable reduction in the expression levels of MUC2, FCGBP, and CLCA1 in the jejunum tissues of PEDV-infected piglets compared to non-infected tissues (Figures 1D–F).

We also performed IHC staining for PEDV-N and MUC2 to evaluate MUC2 levels in the jejunum following PEDV infection (Figure 1G). It was observed that the levels of MUC2 in the intestinal villi significantly decreased after PEDV infection. These findings suggest that PEDV infection results in a decrease in both the number of goblet cells and the mucin content in the intestines.

### PEDV infection modulates Notch, MAPK, and Wnt/β-catenin pathways in a piglet’s small intestine

**Figure 2:**
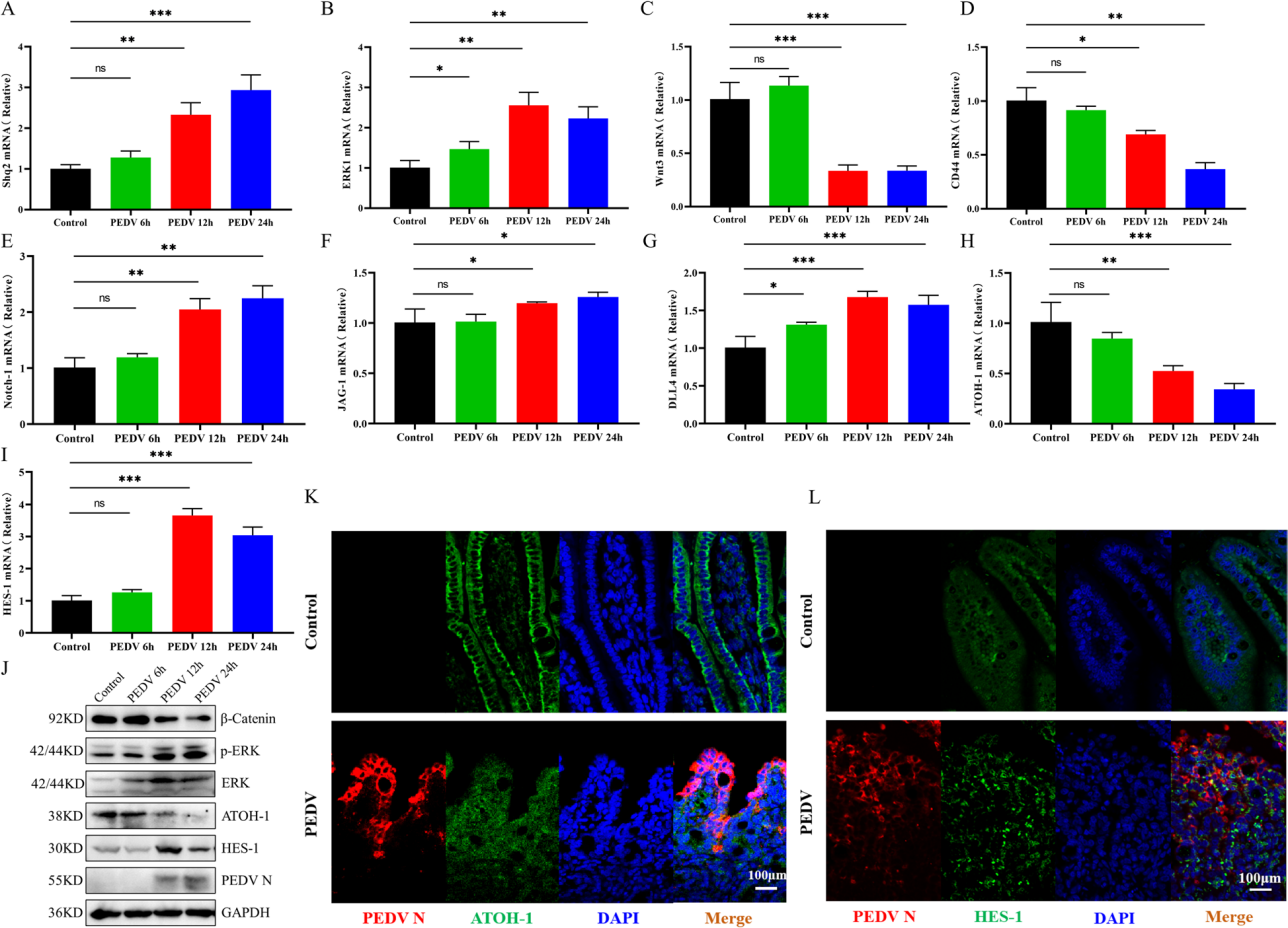
**The regulatory effects of PEDV infection on the Notch, MAPK, and Wnt/β-catenin pathways in the small intestine of piglets.** **A** and **B** mRNA levels of Shp2 and the MAPK effector kinase ERK1 in homogenised jejunum tissues. **C** and **D** mRNA expression of Wnt3 and Wnt target gene CD44 in homogenised jejunum tissues. **E**–**G** mRNA levels of Notch-1, JAG-1 and DLL4 in homogenized jejunum tissues. **H** and **I** ATOH-1 and HES-1 mRNA levels in homogenised jejunum tissues. **J** Western blot analyses of β-catenin, ERK1/2, p-ERK1/2, ATOH-1 and HES-1 proteins in the jejunum of uninfected and PEDV-infected piglets. **K** ATOH-1 (green) and PEDV (red) positive cells in jejunum tissues. **L** HES-1 (green) and PEDV (red) positive cells in the jejunum tissues. Scale bar = 100 μm. *p < 0.05, **p < 0.01, ***p < 0.001.

To clarify the mechanisms behind PEDV-induced goblet cell depletion and defects in mucus secretion, we examined changes in the expression and activity of effectors involved in ISG differentiation following viral infection. As illustrated in Figures 2A and B, the transcription levels of the non-receptor tyrosine phosphatase Shp2, which modulates the activity of MAPK signalling pathways [25], and the MAPK effector kinase ERK1 were significantly elevated in the jejunum of PEDV-infected piglets. Western blot analysis of ERK1/2 and phosphorylated ERK1/2 further confirmed the enhanced activity of the MAPK pathway in the infected jejunum (Figure 2J).

Subsequent expression analyses of Wnt3, β-catenin, and the Wnt target gene CD44 indicated suppressed activity of the Wnt/β-catenin pathway in the infected intestines (Figures2C, D, J). Notch signalling was evaluated by quantifying the mRNA expressions of Notch ligands (JAG-1, DLL-4) and the receptor Notch-1 in the jejunum using RT-qPCR, revealing upregulated expression in infected piglets compared to controls (Figures 2E–G).

Moreover, PEDV infection downregulated ATOH-1, a master transcription factor that directs ISC differentiation toward goblet cells, while upregulating HES-1 protein expression (Figures 2H–J). IHC staining corroborated the reduced ATOH-1 and elevated HES-1 expression in the infected jejunum (Figures 2K–L).

Collectively, these results demonstrate that PEDV infection concurrently activates the Notch and MAPK pathways while suppressing the Wnt/β-catenin pathway in the piglet intestine. Although elevated MAPK and diminished Wnt/β-catenin signalling typically promote goblet cell expansion, the villi of PEDV-infected piglets exhibited goblet cell depletion. This paradox underscores the dominant role of Notch activation in overriding these signals to suppress the differentiation of ISCs into goblet cells.

### Development of 3D intestinal organoids and susceptibility to PEDV in 2D monolayers

Intestinal organoids are 3D multicellular structures that have the ability to self-renew and self-organise. These organoids closely resemble the cell types, structures and functions of the organs or tissues from which they originate [27, 30, 31].

**Figure 3:**
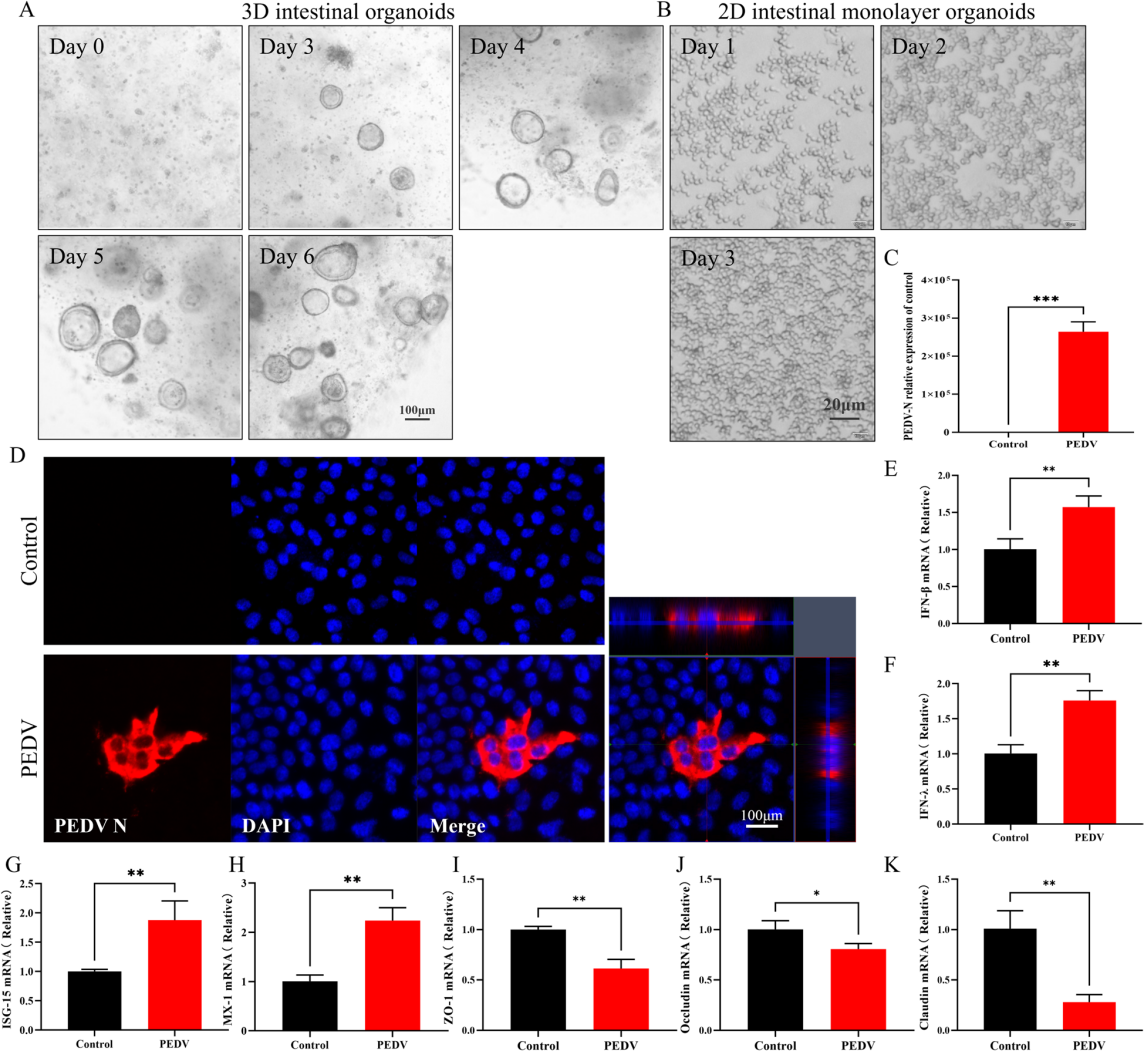
**Porcine intestinal monolayer organoids are susceptible to PEDV infection.** **A** Development of porcine intestinal organoids from day 0 to day 6 as observed by light microscopy. Scale bar = 100 μm. **B** Growth of 2D intestinal monolayer organoids from day 1 to day 3 as observed by light microscopy. Scale bar = 20 μm. **C** RT-qPCR quantified viral replication in intestinal monolayer organoids at 24 hpi. **D** IHC assays of 2D intestinal monolayer organoids infected by PEDV (MOI 1) for 24 h. Scale bar = 100 μm. **E**–**H** The mRNA levels of IFN-β, IFN-λ, and interferon-stimulated genes ISG-15 and MX-1 from uninfected and PEDV-infected (MOI 1) intestinal monolayer organoids. **I**–**K** The mRNA levels of tight-junction-related genes, ZO-1, occludin, and claudin, from uninfected and PEDV-infected intestinal monolayer organoids. *p < 0.05, **p < 0.01, ***p < 0.001.

To study the effects of PEDV infection on intestinal homeostasis, jejunal crypts were isolated from the porcine small intestine and cultured to form 3D intestinal organoids, following the approach outlined by van der Hee et al. [27]. During culture, the crypts underwent progressive differentiation, ultimately forming spherical organoids (Figure 3A) with a central lumen surrounded by an epithelial layer that featured villus-like projections and crypt-like domains.

To further validate the impact of PEDV infection on the functional properties of these intestinal structures, we generated 2D monolayers derived from 3D jejunal organoids, following the method outlined by van der Hee et al. (Figure 3B) [27]. Porcine intestinal monolayer tissue was collected at 24 hpi with PEDV (MOI 1). RT-qPCR and IF analyses indicated that the porcine intestinal monolayer organoids were susceptible to PEDV infection (Figures 3C and D).

To investigate the immune responses of intestinal monolayer organoids to PEDV infection, we measured the expression levels of type I and III interferons. The results were consistent with findings from in vivo studies, showing significant upregulation of IFN-β and IFN-λ in PEDV-infected organoids (Figures 3E and F). Additionally, PEDV infection significantly induced the expression of ISGs, including key markers MX-1 and ISG-15 (Figures 3G and H). Furthermore, PEDV infection resulted in reduced expression of tight junction-associated genes, such as ZO-1, claudin, and occludin, in the intestinal monolayer (Figures 3I–K). In conclusion, this study confirms that the intestinal monolayer organoids can serve as a susceptible model for PEDV infection, effectively simulating the virus replication process and reproducing the pathological characteristics observed in vivo.

### PEDV activates the Notch signalling pathway, reducing goblet cells and mucus secretion in intestinal organoids

**Figure 4:**
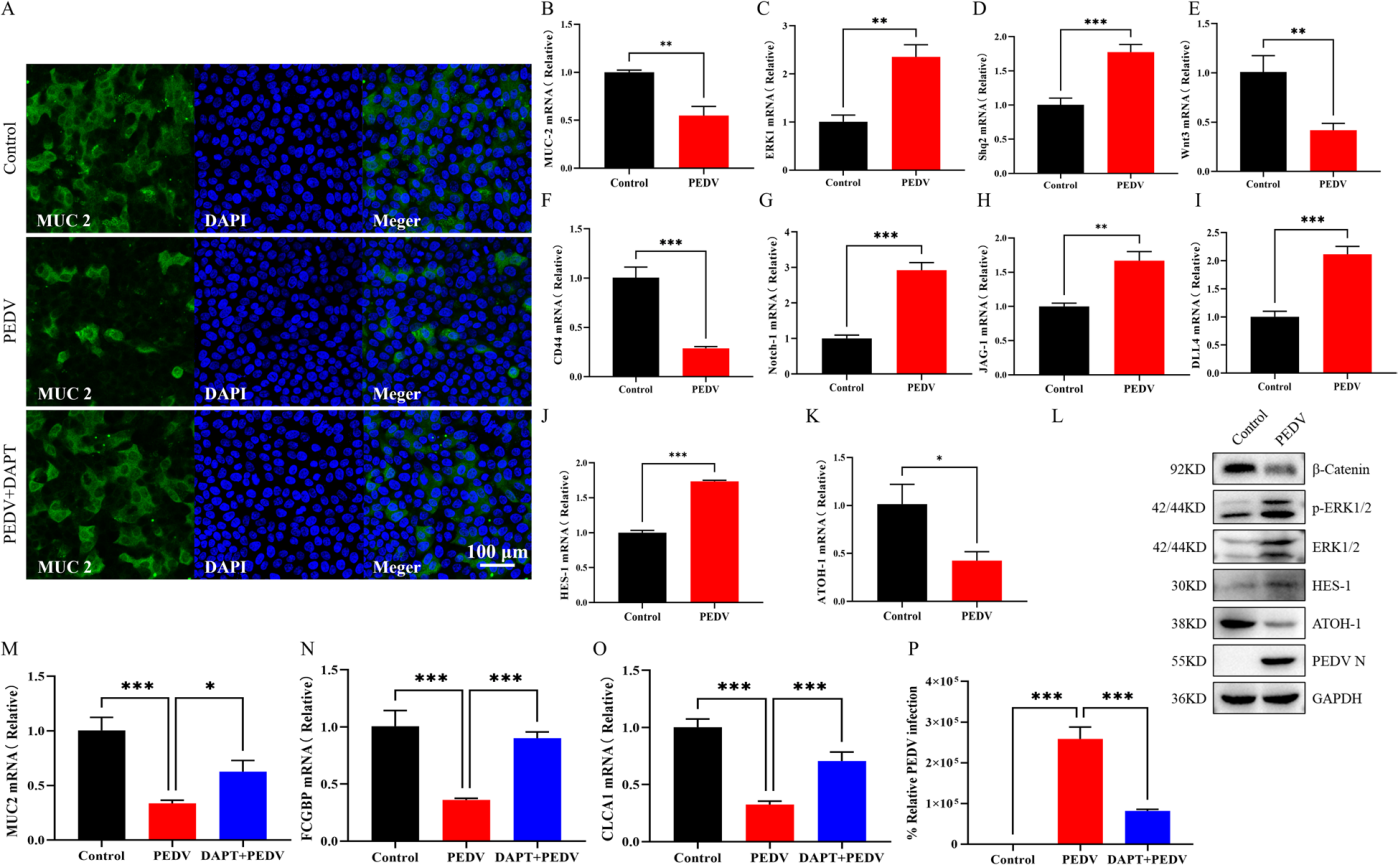
**PEDV activates the Notch signalling pathway, reducing goblet cells and mucus secretion in the intestinal organoids.** **A**) Infected, uninfected and DAPT plus PEDV-treated monolayer organoid cells were stained with IFA; the MUC2-positive cells indicate the goblet cells. Scale bar = 100 μm. (**B**) MUC-2 mRNA levels from the intestinal monolayer organoids. (**C**, **D**) mRNA expression of Shp2 and the MAPK effector kinase ERK1 in uninfected and PEDV-infected monolayer organoids. (E–F) Wnt3 and Wnt target gene CD44 mRNA levels in uninfected and PEDV-infected monolayer organoids. (**G**–**K**) Notch-1, JAG-1, DLL-4, HES-1, and ATOH-1 mRNA levels in uninfected and PEDV-infected intestinal monolayer organoids. (**L**) Western blot analyses of β-catenin, ERK1/2, p-ERK1/2, HES-1 and ATOH-1 expression levels in infected and uninfected monolayer organoids. (**M**–**O**) MUC2, FCGBP, and CLCA1 mRNA levels of the infected and DAPT-treated (10 μM/L) monolayer organoids. (**P**) PEDV-N mRNA levels assessed by RT-qPCR. *p < 0.05, **p < 0.01, ***p < 0.001.

The impact of PEDV infection on goblet cells was further evaluated using intestinal organoid models. Consistent with the in vivo findings, PEDV-infected organoids showed a significant decrease in the number of goblet cells (Figure 4A). Additionally, the level of MUC2 was markedly reduced (Figure 4B).

We also investigated the activities of the MAPK and Wnt/β-catenin pathways within the organoids. Similar to the in vivo results, PEDV infection activated the MAPK pathway while suppressing the Wnt/β-catenin signalling in piglet intestinal organoids (Figures 4C–F, L).

To further confirm the regulatory effect of PEDV infection on the Notch signalling pathway in intestinal organoids, we assessed the expression levels of key molecules. The results indicated that the levels of Notch-1, JAG-1, and DLL4 mRNA were significantly elevated compared to the uninfected controls (Figures 4G–I). Furthermore, HES-1 expression was markedly higher, while ATOH-1 expression was significantly lower in PEDV-infected intestinal monolayer organoids (Figures 4J–L).

The Notch signalling inhibitor DAPT was used to prevent goblet cell loss in PEDV-infected intestinal monolayer organoid systems. As shown in Figure 4A, DAPT-treated PEDV-infected organoids had a higher number of goblet cells compared to untreated infected organoids. Additionally, the levels of MUC2, FCGBP, and CLCA1 were significantly increased in the DAPT-treated PEDV-infected organoids (Figures 4M–O). Furthermore, inhibiting the Notch pathway significantly reduced PEDV infection compared to the control group, suggesting that PEDV may promote infection by activating the Notch pathway (Figure 4P). These results indicate that PEDV infection leads to goblet cell loss by activating the Notch signalling pathway in both in vitro and in vivo models.

### PEDV-encoded ORF3 protein activates the Notch signalling pathway in 2D monolayer organoids

To determine which PEDV-encoded protein activates the Notch signalling pathway, preliminary screening was conducted using HEK 293 T cells transfected with high expression levels of PEDV-encoded proteins (NSP1-NSP10, NSP15, S, ORF3, M, and N) [32]. mRNA levels of Notch-1, HES-1, and ATOH-1 were measured, revealing that ORF3 induced the most significant activation of the Notch signalling pathway (Additional file 3).

**Figure 5:**
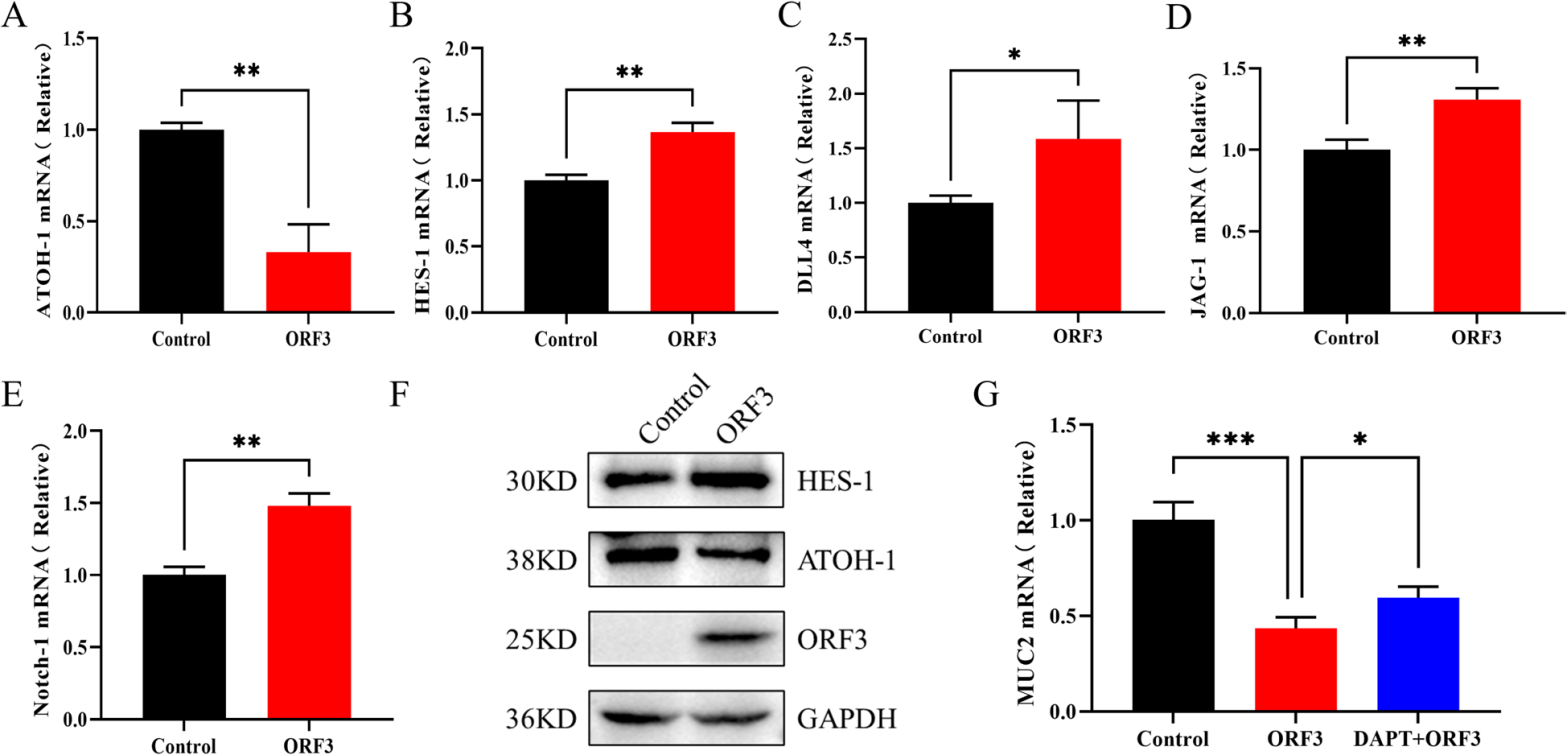
**Notch signalling is activated in intestinal monolayer organoids by overexpression of PEDV ORF3 protein.** (**A**–**E**) ATOH-1, HES-1, DLL4, JAG-1, and Notch-1 mRNA levels in control and PEDV-ORF3-transfected monolayer organoids. (**F**) Western blot analyses of ATOH-1 and HES-1 proteins in control and ORF3-transfected monolayer organoids. (**G**) MUC-2 mRNA levels of ORF3-transfected and DAPT-treated (10 μM/L) monolayer organoids. *p < 0.05, **p < 0.01, ***p < 0.001.

Following this, the ORF3 expression plasmid was transiently transfected into intestinal monolayer organoids. RT-qPCR analysis demonstrated that ORF3 overexpression significantly upregulated the mRNA levels of JAG-1, DLL4, Notch-1, and HES-1, while simultaneously downregulating ATOH-1 (Figures 5A–E). At the protein level, ORF3 also enhanced HES-1 expression and decreased ATOH-1 expression (Figure 5F). Furthermore, ORF3 transfection reduced MUC2 expression; however, DAPT treatment reversed this ORF3-induced reduction (Figure 5G).

These findings indicate that the PEDV-encoded ORF3 protein activates the Notch signalling pathway, leading to the inhibition of mucus production in monolayer organoids.

## Discussion

PEDV primarily infects the villous epithelial cells in the porcine small intestine, leading to villous atrophy and dysfunction of the intestinal barrier. This infection results in structural damage to the intestine, including the destruction of epithelial cells and malabsorption, ultimately causing diarrhoea [11, 33].

The villi and crypts make up the intestinal epithelial tissue, and the renewal and homeostasis of the intestinal epithelium are achieved through self-renewal in the crypt region [34]. In this study, we observed that in the jejunum of piglets, the viral load of PEDV peaked at 12 hpi and significantly decreased by 24 hpi. During this time, PEDV infection downregulated the expression of tight junction-related proteins (occludin, claudin, and ZO-1), resulting in severe villous atrophy and crypt hyperplasia. These findings indicate that PEDV efficiently infects the small intestines of piglets, causing rapid mucosal damage to the intestinal villi.

Although the host initiated innate defensive responses by elevating the expression of IFNs and ISGs, these responses were insufficient to prevent the rapid replication of PEDV or to mitigate the intestinal injury effectively.

Mucin molecules are essential for maintaining the structural integrity of mucus, providing physical protection through the mucus barrier [35]. The intestinal mucus layer plays a crucial role in ensuring intestinal stability and safeguarding against external threats [29, 36]. In the small intestine, the primary secreted mucus protein is MUC2 [37], which is mainly produced by intestinal goblet cells [38].

Previous studies have indicated that gut pathogens can impact the formation and function of goblet cells. For instance, enterovirus 71 [39], human astrovirus VA1 species [40], and murine astrovirus (MuAstV) [41] all exhibit a tropism for goblet cells and alter their function. It has been established that PEDV can infect various cell types, including enterocytes, stem cells, and goblet cells [42]. In prior research, we found that PEDV infection of intestinal goblet cells in piglets significantly reduced MUC2 protein expression in these cells [43].

In this study, we further demonstrated that PEDV infection disrupts the differentiation of goblet cells in the jejunum of piglets, leading to a sustained reduction in MUC2 expression. These findings suggest that PEDV infection compromises the function of ISCs and disrupts the intestinal mucus barrier.

Additionally, we observed the dynamics of the virus in the jejunal segments of infected piglets, noting that the viral load peaked at 12 hpi and declined by 24 hpi. However, MUC2 expression levels and goblet cell numbers exhibited a continuous decrease throughout the infection period (6–24 hpi). This persistent decline may reflect cumulative damage caused by viral replication despite decreasing titres.

The MAPK, Wnt/β-catenin, and Notch pathways play crucial roles in the differentiation of ISCs. MAPK signalling influences the decision between goblet and Paneth cell differentiation by modulating Wnt/β-catenin signalling [25]. Recent studies have shown that PEDV infection activates both the MAPK and Wnt/β-catenin pathways in various in vitro cell lines [43, 44]. In contrast, our study demonstrates that PEDV infection activates MAPK pathways while simultaneously suppressing Wnt/β-catenin signalling in the intestines of piglets and in intestinal organoids.

This alteration in signalling can enhance the differentiation of ISCs into goblet cells. However, we observed a significant reduction in the number of goblet cells in the small intestines of piglets and in intestinal organoids infected with PEDV. This reduction coincided with a notable activation of the Notch pathway during the viral infection. Our findings suggest that the Notch activation induced by plays a crucial role in inhibiting goblet cell differentiation, counteracting the potential pro-goblet effects of the altered MAPK and Wnt signalling. As a result, goblet cells are not effectively replenished, and the mucus layer fails to be repaired promptly after the infection. This situation facilitates the viral invasion of intestinal epithelial cells and exacerbates intestinal damage.

In our investigation of viral proteins that regulate the Notch pathway, we identified that the PEDV-encoded protein ORF3 significantly activates Notch signalling. ORF3 is a multifunctional viral protein that plays a crucial role in cellular regulation, particularly in immune responses and apoptosis [45].

Accumulating evidence shows that ORF3 is involved in various cellular processes: it prolongs the DNA synthesis phase (S-phase) and enhances vesicle formation [13]. This extension of the S-phase leads to a reduction in cell proliferation and an increase in cell death, ultimately delaying the onset of differentiation [46].

Importantly, the activation of Notch signalling also delays S-phase entry and induces cell cycle arrest [47]. This suggests a potential link between ORF3-induced Notch activation and its role in regulating the cell cycle.

This study clearly shows a strong correlation between PEDV infection and a reduction in goblet cells. The infection led to villus atrophy, crypt hyperplasia, downregulation of tight junction-related proteins, and significant damage to intestinal integrity. Furthermore, PEDV infection caused a loss of goblet cells by activating the Notch signalling pathway and reducing the expression of MUC2 in the intestinal tract. This impairment diminishes the intestinal repair capacity, exacerbates the viral infection, and leads to further intestinal damage. Overall, these findings provide valuable insight into the mechanisms behind PEDV-induced intestinal pathology.

## Supplementary Information

**Additional file 1: The table of primer sequences for RT-qPCR.**

**Additional file 2: PEDV infection disrupts the intestinal barrier.** (A) PEDV-N protein in the jejunum segment of infected and uninfected piglets. (B) H&E staining of jejunum segments of uninfected and infected piglets. The black arrow indicates a damaged villus. Scale bar: 50 μm. (C and D) The measures of villus height and crypt depth, and the villus/crypt ratios of jejunum segments of uninfected and infected piglets. (E-H) IFN-λ, IFN-β, MX-1, and ISG-15 mRNA in jejunum tissues. (I-K) ZO-1, occludin, and claudin mRNA levels in jejunum tissues. *, *p* < 0.05, **, *p* < 0.01, ***, *p* < 0.001.

**Additional file 3: ORF3 activates the Notch pathway. **(A) WB detections of PEDV-encoded proteins in HEK-293T cells. (B-D) Notch-1, HES-1, and ATOH-1 mRNA levels in PEDV-encoded proteins transiently transfected HEK-239T cells.

## Data Availability

All data used for statistical analyses and to generate graphs have been uploaded to the Dryad dataset [48].
