# Supplementary material for: PEDV infection downregulates goblet cell differentiation through activating the Notch pathway
Source: Vet Res. 2025 Aug 12;56:168. doi: 10.1186/s13567-025-01599-5 (PMC12341102; doi:10.1186/s13567-025-01599-5)
Supplement: Supplementary file 1 — Additional file 1: The table of primer sequences for RT-qPCR. [file 13567_2025_1599_MOESM1_ESM.docx]

| Species | Primers | Sequence (5′ - 3′) | Accession |
| --- | --- | --- | --- |
| Sus scrofa | FCGBP F | CGGTACTTACCGGCGAACTC | XM_021094376.1 |
|  | FCGBP R | CCCAGCCTGAATCTCGCATA |  |
|  | CLCA1 F | GTTCCGAAGTCTCCCGTACC | NM_214148.3 |
|  | CLCA1 R | TGATGTACCTGTCAGCTCGC |  |
|  | NOTCH-1 F | TGCAGAGTGAGACGGTGGA | XM_021081037.1 |
|  | NOTCH-1 R | AGAAGAGCAGCACGAAGGC |  |
|  | HES-1 F | TACCCCAGCCAGTGTCAA | NM_001195231.1 |
|  | HES-1 F | AATGTCCGCCTTCTCCAG |  |
|  | GAPDH F | CCTCCCCGTTCGACAGAC | NM_001206359.1 |
|  | GAPDH R | ATGCGGCCAAATCCGTT |  |
|  | IFN-β F | TGCATCCTCCAAATCGCTCT | NM_001003923.1 |
|  | IFN-β R | ATTGAGGAGTCCCAGGCAAC |  |
|  | IFN-λ F | GAGGCCCCCAAAAAGGAGT | NM_001142837.1 |
|  | IFN-λ R | AGGTTCCCATCGGCCACATA |  |
|  | ISG-15 F | ACAGCCATGGGCTGGGA | NM_001128469.3 |
|  | ISG-15 R | CCTTCAGCTCTGACACCGAC |  |
|  | MX-1 F | ACAGAACCGCCAAGTCCAAA | NM_214061.2 |
|  | MX-1 R | GCGGATCAGCTTCTCACCTT |  |
|  | ZO-1 F | GAAATACCTGACGGTGCTGC | XM_021098856.1 |
|  | ZO-1 R | GAGGATGGCGTTACCCACAG |  |
|  | Occludin F | TTGCGGCGAGCGGATTG | NM_001163647.2 |
|  | Occludin R | GCCTGGATGACATGGCTGAT |  |
|  | Claudin F | CCAGTCAATGCCAGGTACGA | NM_001244539.1 |
|  | Claudin R | CAAAGTAGGGCACCTCCCAG |  |
|  | JAG-1 F | CGACCCCCTGTGAAGTGATT | XM_005672699.3 |
|  | JAG-1 R | ACTCTTGCACTTCCCGTGAG |  |
|  | HES-1 F | AGCATCTGAGCACAGAAAGTCA | NM_001195231.1 |
|  | HES-1 R | GGAATGCCGCGAGCTATCTT |  |
|  | ATOH-1 F | CACGGGCTGAACCACGCCTT | XM_003129319.4 |
|  | ATOH-1 R | GGTACCCGCGCTTGCTTCGT |  |
|  | MUC2 F | CTGCTATGTCGAGGACACCC | XM_021082584.1 |
|  | MUC2 R | GAGTTGGTACACACGCAGGA |  |
|  | DLL4 F | CCCAGGGACTCCATGTACCA | NM_001244418.1 |
|  | DLL4 R | GCTCCTGCCTTATACCTCCG |  |
|  | PEDV N F | CGCAAAGACTGAACCCACTAACTT | KU646831.1 |
|  | PEDV N R | TTGCCTCTGTTGTTACTCGGGGAT |  |
| Human | MUC2 F | CTGCTATGTCGAGGACACCC | NM_002457.5 |
|  | MUC2 R | GAGTTGGTACACACGCAGGA |  |
|  | GAPDH F | AATGAATGGGCAGCCGTTAG | NM_001256799.3 |
|  | GAPDH R | GCAGGAGGCATTGCTGATGAT |  |
|  | Notch-1 F | GTGCCCTGGGCTTCTCTG | NM_017617.5 |
|  | Notch-1 R | GGCACGATTTCCCTGACCA |  |
|  | DLL4 F | CCCAGGGACTCCATGTACCA | NM_019074.4 |
|  | DLL4 R | GCTCCTGCCTTATACCTCCG |  |
|  | ATOH-1 F | CAGCTGCGCAATGTTATCCC | NM_005172.2 |
|  | ATOH-1 R | TTGTAGCAGCTCGGACAAGG |  |
|  | JAG-1 F | CGACCCCCTGTGAAGTGATT | NM_000214.3 |
|  | JAG-1 R | ACTCTTGCACTTCCCGTGAG |  |
|  | HES-1 F | ACGACACCGGATAAACCAAAGA | NM_005524.4 |
|  | HES-1 R | ATGCCGCGAGCTATCTTTCT |  |
