# Supplementary figures and images for: PEDV infection downregulates goblet cell differentiation through activating the Notch pathway
Source: Vet Res. 2025 Aug 12;56:168. doi: 10.1186/s13567-025-01599-5 (PMC12341102; doi:10.1186/s13567-025-01599-5)

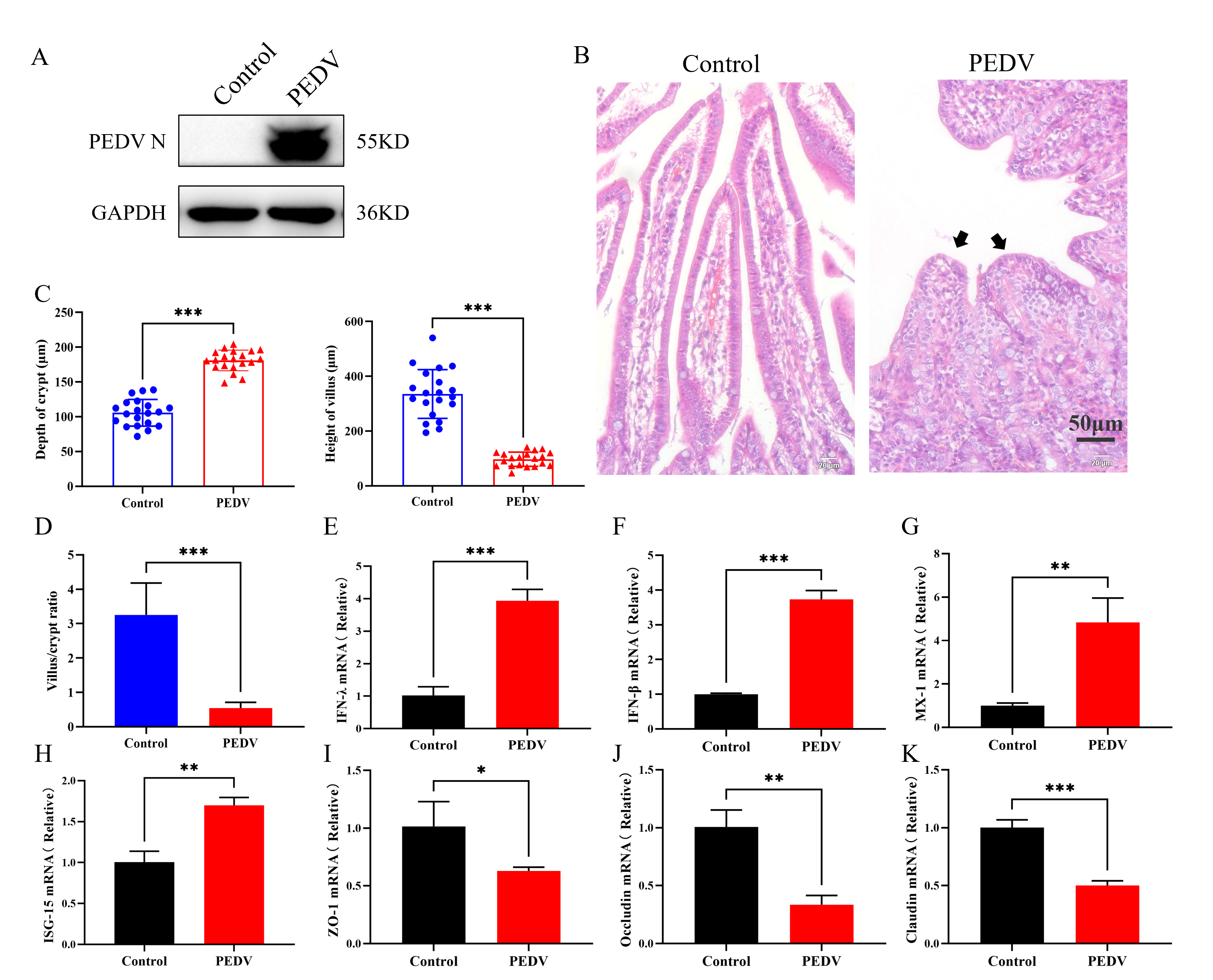

Supplement: Supplementary file 2 — Additional file 2: PEDV infection disrupts the intestinal barrier. (A) PEDV-N protein in the jejunum segment of infected and uninfected piglets. (B) H&E staining of jejunum segments of uninfected and infected piglets. The black arrow indicates a damaged villus. Scale bar: 50 μm. (C and D) The measures of villus height and crypt depth, and the villus/crypt ratios of jejunum segments of uninfected and infected piglets. (E-H) IFN-λ, IFN-β, MX-1, and ISG-15 mRNA in jejunum tissues. (I-K) ZO-1, occludin, and claudin mRNA levels in jejunum tissues. *, p < 0.05, **, p < 0.01, ***, p < 0.001. [file 13567_2025_1599_MOESM2_ESM.tif]

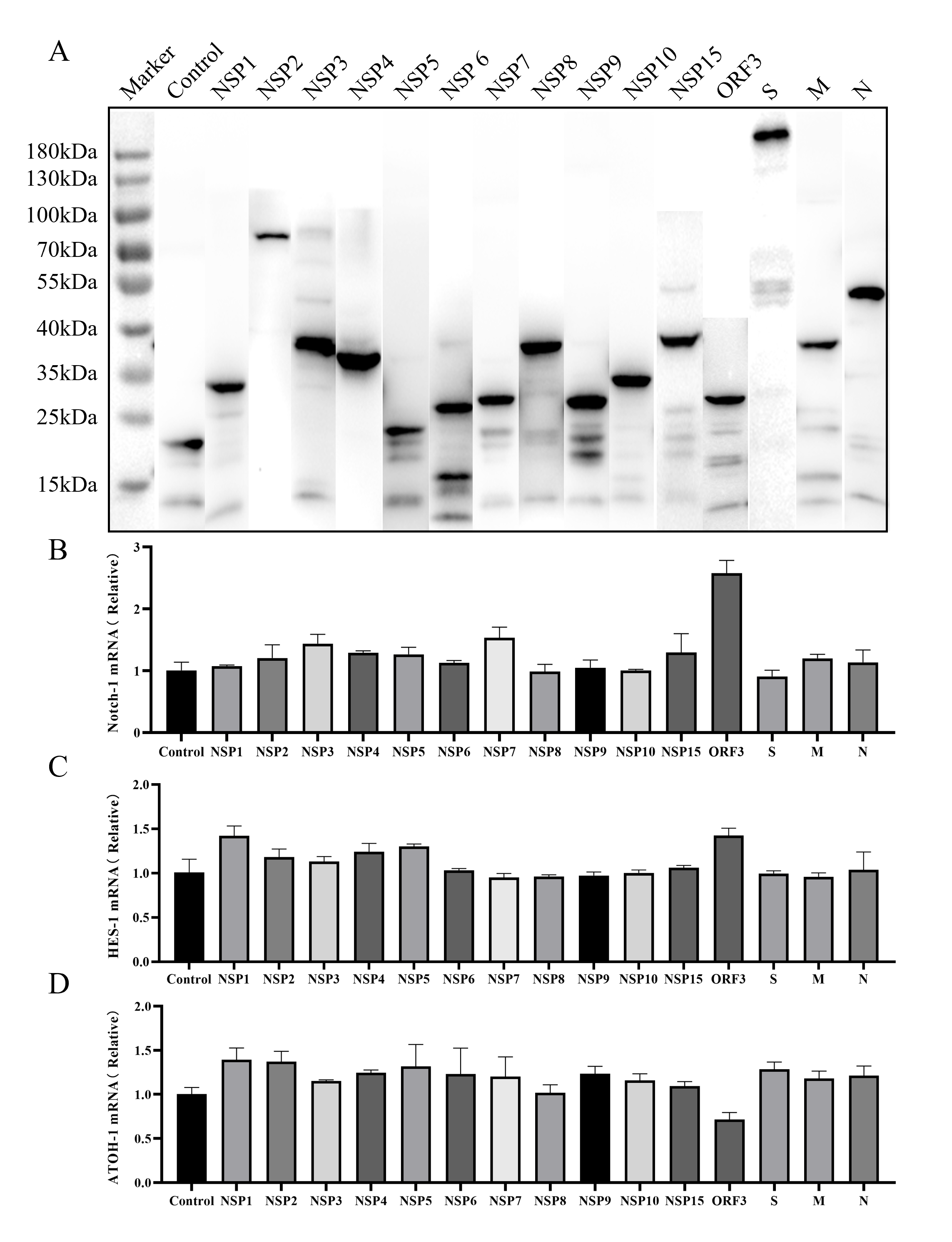

Supplement: Supplementary file 3 — Additional file 3: ORF3 activates the Notch pathway. (A) WB detections of PEDV-encoded proteins in HEK-293T cells. (B-D) Notch-1, HES-1, and ATOH-1 mRNA levels in PEDV-encoded proteins transiently transfected HEK-239T cells. [file 13567_2025_1599_MOESM3_ESM.tif]
